# Microbiota-derived metabolites in tumorigenesis: mechanistic insights and therapeutic implications

**DOI:** 10.3389/fphar.2025.1598009

**Published:** 2025-05-15

**Authors:** Si-Fu Yang, Xiao-Chen Chen, Yao-Jie Pan

**Affiliations:** Cancer Center, Department of Medical Oncology, Zhejiang Provincial People’s Hospital, Affiliated People’s Hospital, Hangzhou Medical College, Hangzhou, Zhejiang, China

**Keywords:** microbiota, metabolites, tumorigenesis, signaling/signaling pathways, metabolite

## Abstract

Intestinal microbiota is a complex ecosystem of microorganisms that perform diverse metabolic activities to maintain gastrointestinal homeostasis. These microorganisms provide energy and nutrients for growth and reproduction while producing numerous metabolites including lipopolysaccharides (LPS), *Bacteroides* fragilis toxin (BFT), bile acids (BAs), polyamines (PAs), and short-chain fatty acids (SCFAs). These metabolites are linked to inflammation and various metabolic diseases, such as obesity, type-2 diabetes, non-alcoholic fatty liver disease, cardiometabolic disease, and malnutrition. In addition, they may contribute to tumorigenesis. Evidence suggests that these microbes can increase the susceptibility to certain cancers and affect treatment responses. In this review, we discuss the current knowledge on how the gut microbiome and its metabolites influence tumorigenesis, highlighting the potential molecular mechanisms and prospects for basic and translational research in this emerging field.

## 1 Introduction

The human microbiota consists of over 100 trillion organisms, including bacteria, viruses, fungi, and protozoans, which primarily reside on the epithelial surfaces of the human body. The human gut provides nutrient-rich and livable conditions for the microbiome. The gut microbiota benefits the human body by producing various metabolites such as short-chain fatty acids (SCFAs) from dietary fiber, synthesizing vitamins B and K, metabolizing compounds such as sterols and xenobiotics, and performing immunoregulatory functions (Rooks and Garrett, 2016). Its role in diseases such as cancer, liver disease, obesity, and neuropsychiatric disorders has been increasingly recognized (Garrett, 2015; Hand et al., 2016). Intestinal microbiota metabolites have been evaluated not only for their impact on the onset and progression of different tumor types but also for their potential as biomarkers and cancer therapies (Yu and Schwabe, 2017; Fernandez et al., 2018).

**FIGURE 1:**
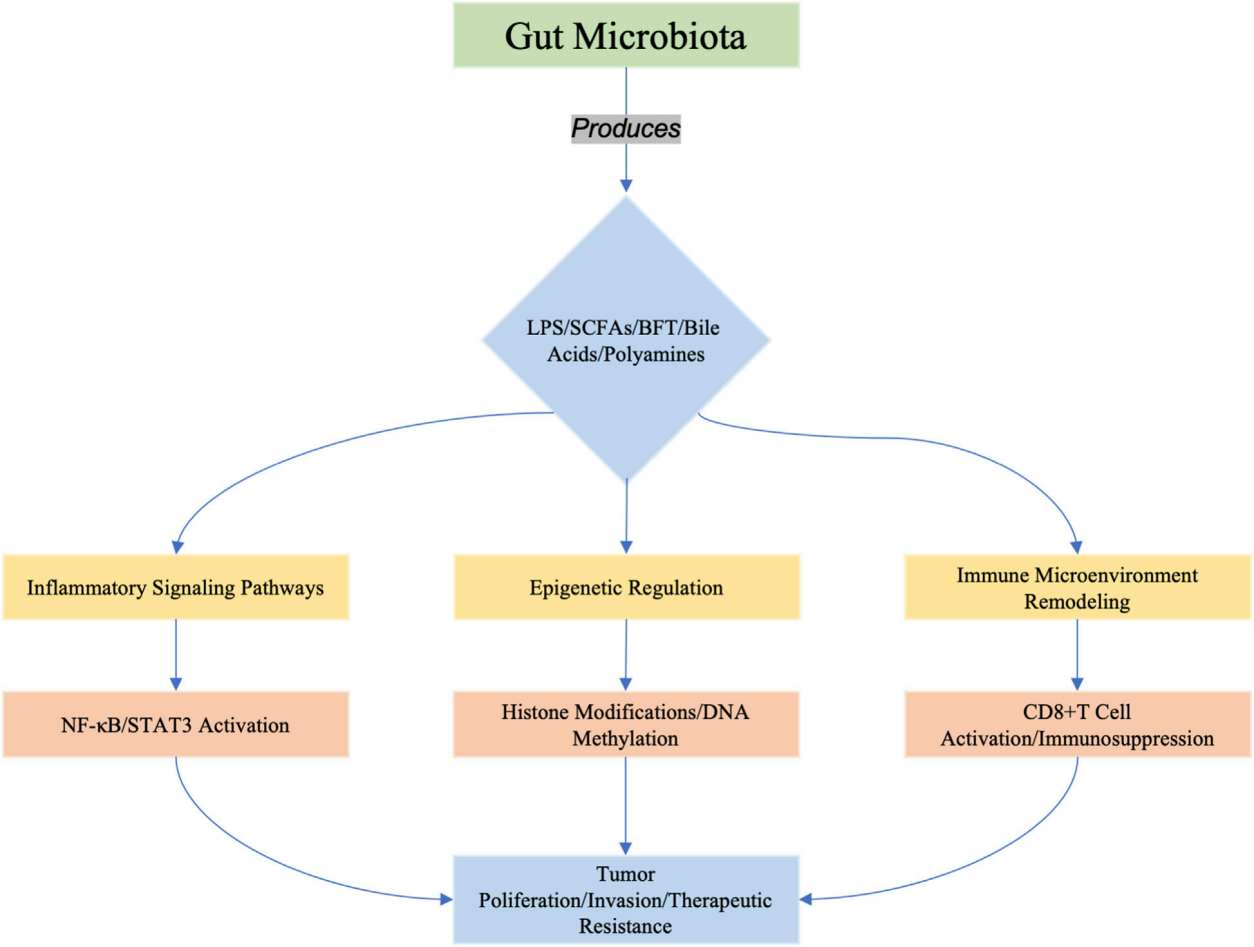
Microbiota-derived metabolites (e.g., LPS, SCFAs, BFT, bile acids, and polyamines) orchestrate tumorigenesis through three interconnected axes: (1) inflammatory signaling (e.g., TLR4/NF-κB activation), (2) epigenetic reprogrammimg (e.g., histone acetylation/DNA methylation), and (3) immune microenvironment modulation (e.g., enhancing immunosurveillance or promoting immunosuppression). These pathways converge to drive tumor cell proliferation, invasion, and resistance to therapy. Recent studies highlight emerging roles of metabolites in shaping cancer stemness (e.g., BFT-induced Notch/β-catenin activation in breast cancer) and metabolic reprogramming (e.g., butyrate-mediated epigenetic regulation in HCC).

This review systematically dissects the dual roles of microbiota-derived metabolites in tumorigenesis, focusing on their bidirectional interactions with oncogenic signaling, immune microenvironment, and therapeutic responses (Figure 1). Recent breakthroughs in precision oncology and metabolomics are highlighted to bridge basic science with clinical translation.

## 2 Microbiota-derived metabolites and oncogenic pathways

### 2.1 Lipopolysaccharide (LPS)

Gram-negative bacterial LPS is a major component of the outer membrane and plays a key role in host-pathogen interactions with the innate immune system (Maldonado et al., 2016). By activating the transcription factor NF-κB and other cytokines, bacteria-secreting LPS can trigger the host immune response through a cascade of LPS receptors, such as Toll-like receptor 4 (TLR4) and cluster of differentiation 14 (CD14), leading to an inflammatory or immunomodulatory environment. In this study, we focused on the effects and mechanisms of LPS on tumorigenesis.

In colorectal carcinoma (CRC), exposure to LPS increases the expression of CXC chemokine receptor 7 (CXCR7) and enhances the proliferation and migration of SW480 and Colo 205 cells via the TLR4/myeloid differential protein (MD-2) pathway (Xu et al., 2011). Kuo et al. reported that normal human colonocytes are CD14^+^TLR4^−^, whereas cancerous tissues are CD14^+^TLR4^+^. In the absence of TLR4, LPS mediates colonocyte apoptosis by binding to CD14, which is dependent on CD14-mediated lipid messengers and PKC phosphorylation. In contrast, in CD14^+^TLR4^+^ cells, apoptosis can be blocked by competitive antagonism of TLR4 binding to LPS, leading to cancer progression (Kuo et al., 2015). Another study demonstrated that LPS augments VEGF-C secretion to promote cell motility and lymphangiogenesis via TLR4-NF-κB/JNK signaling (Zhu et al., 2016a). LPS can also bind to the gene promoter of VEGFR-3 to facilitate CRC migration and invasion via this signaling axis (Zhu et al., 2016b). Additionally, LPS promotes proliferation by facilitating the mRNA expression of inflammatory cytokines such as COX-2, IL-6, iNOS, and TNF-α (Suh et al., 2019; Hsieh et al., 2016). LPS has emerged as a powerful regulator of CRC tumorigenesis, attracting widespread attention and interest from researchers, leading to the development of countermeasures. In 2018, Song et al. engineered an LPS-targeting fusion protein by loading its coding sequence into a lipid-protamine-DNA (LPD) nanoparticle system for selective expression of the LPS trap protein, blocking LPS inside the tumor. This nanotrapping system significantly relieved the immunosuppressive microenvironment and boosted anti-PD-L1 mAb therapy against CRC tumors (Song et al., 2018). Despite its promise for cancer immunotherapy, the strong proinflammatory properties of LPS result in severe localized and systemic side effects, limiting its administrable dosage and potential for chronic dosing. Boushehri et al. further improved this nanotrapping system and found that size was an important determinant of short-term tolerability, with larger particles being associated with a higher incidence and extent of localized necrosis. In contrast, nanostructure composition predominantly governs long-term systemic tolerability. The higher affinity of LPS molecules for the triglyceride core of the nanoemulsion compared to that of the polymeric matrix significantly improves the tolerability of the former over time (Shetab Boushehri et al., 2019).

In hepatocellular carcinoma (HCC), LPS promotes cell survival, proliferation, invasion, and production of pro-inflammatory mediators, including TNF-α, iNOS, IL-1β, IL-6, CCL-2, CCL-22, vimentin, and epidermal growth factor receptor (EGFR), through the induction of TLR4 signaling (Wang et al., 2013; Lo et al., 2018). Singh et al. found that Nox4 mediates LPS-TLR4 signaling in human hepatoma cells, potentially contributing to LPS-induced liver pathology (Singh et al., 2017). Another study has shown that LPS antagonizes the inhibitory effect of miR-145 via NF-κB p65 activation (Wang RK. et al., 2019). Additionally, LPS was found to enhance HCC migration and invasion by targeting HIF-1 via NF-κB. Thus, we speculated that the LPS-TLR4-NF-κB axis might play a crucial role in HCC tumorigenesis, similar to that in CRC. LPS can also increase IL-1β production via protein kinase R, thereby enhancing HCC proliferation and invasion (Imai et al., 2019). Another key target of LPS is STAT3, which facilitates HCC cell proliferation, migration, and angiogenesis (Wang Z. et al., 2019). A recent study revealed that LPS increases N6-methyladenosine (m6A) methylation of GNAS mRNA, upregulating protein expression and activating STAT3 and IL-6 production (Zhang et al., 2020), thus outlining the GNAS-LPS-STAT3-IL-6 axis in HCC tumorigenesis. Although several studies have reported that LPS promotes tumor development, Honda et al. discovered that LPS-treated human monocytes may effectively suppress tumor invasion and proliferation in hepatic cancers. The co-cultured human monocyte cell line THP-1 and hepatic cancer cell line HepG2 were treated with LPS, resulting in significant suppression of the mRNA expression of monocyte chemotactic protein-1, vascular endothelial growth factor-A, TNF-α, IL-1β, IL-8, NF-κB, RelB, STAT3, IL-10, and transforming growth factor-β in THP-1 cells (Honda and Inagawa, 2016). Similarly, Spirulina-derived LPS was found to manipulate the balance of the IFN-γ-IL-17/IL-23 axis towards IFN-γ production, suppressing HCC progression. The antitumor activity and IFN-γ production were mediated by T cells. *In vitro* experiments showed that Spirulina LPS impaired the antigen-presenting function, supporting the generation of IL-17-producing cells in a TLR4-dependent manner (Okuyama et al., 2017). This study supports the use of TLR-based immunomodulators in tumor immunotherapy.

In lung cancer (LC), LPS mainly promotes cell proliferation and epithelial-mesenchymal transition (EMT) while attenuates apoptosis (Jiang et al., 2019; Wei et al., 2019). Mechanistically, LPS activates the TLR4 signaling pathway to facilitate immune suppression factors, such as TGF-β, VEGF, and IL-8, aiding the immune escape of cancer cells (Xu et al., 2017). LPS can also bind to CD14 and TLR4, leading to COX-2 activation and subsequent PGE_2_ release (Hattar et al., 2013). We conclude that CD14/TLR4-dependent COX-2 activation is a crucial step in mediating tumor proliferation in response to LPS. Wang et al. found that LPS activates the NLRP3 inflammasome to promote LC cell proliferation and migration (Wang et al., 2016). Further research demonstrated that LPS could be bound by secretoglobin SCGB3A2 and delivered to the cytosol to activate caspase-11/NLRP3 inflammasome foci formation, thereby decreasing cell proliferation (Yokoyama et al., 2018). Despite this bilateral activation of the inflammasome, LPS is more likely to promote cancer cell proliferation when confined to the cell membrane surface rather than suppressing it.

In breast cancer (BC), LPS facilitates EMT and cell metastasis, partly through the TLR4-Akt-GSK3β-β-catenin signaling pathway (Huang et al., 2013; Hong et al., 2015; Yang et al., 2014; Cho et al., 2015). Specifically, LPS stimulation of the TLR4 pathway in MCF7 and MDA-MB-231 breast cancer cells results in the following: (1) promotes of cell migration, (2) activates of the β-catenin signaling pathway via PI3K/Akt/GSK3β, and (3) enhances transcription of β-catenin target genes, leading to metastasis (Li J. et al., 2017). Additionally, Fried et al. demonstrated that the induction of LPS could mediate BC cell apoptosis in an IFN-β-dependent manner (Fried et al., 2016).

In gastric cancer (GC), LPS binds to CD14, increasing cell viability and inflammatory factor production while inhibiting apoptosis (Li et al., 2015). It also promotes STAT3 phosphorylation, which upregulates MMP7, MMP9, and VEGF expression (Guo et al., 2015). By binding to receptors such as TLR1, TLR4, TLR6, CD14, and MD2, LPS activates the NF-κB and STAT3 signaling pathways, inducing the production of TNF-α, IL-6, IL-1β, IL-8, and CXCR7 (Guo et al., 2015).

In glioma, LPS not only upregulates inflammatory mediators such as IL-8, CXCL8, and IL-1β to support tumorigenesis but also alters the immunophenotype of glioma cells and induces antitumor immunity via TLR4 (Li et al., 2015; Braganhol et al., 2015; Han et al., 2017). Hu et al. reported that LPS induces the Notch signaling pathway, activating TLR4, and reversing tumor differentiation (Han et al., 2017).

Emerging evidence suggests that impaired intestinal barrier function facilitates LPS translocation into systemic circulation. Under pathological conditions such as metabolic syndrome or chemotherapy-induced mucositis, increased gut permeability allows LPS to traverse the intestinal epithelium via paracellular transport or through M-cell mediated transcytosis (Ghosh et al., 2020). Once entering the portal circulation, LPS binds to lipopolysaccharide-binding protein(LBP) and is shuttled to CD14/TLR4 receptors on Kupffer cells, establishing a pro-tumorigenic microenvironment in the liver (Tsukamoto et al., 2018). For extrahepatic tumors, circulating LPS may directly activate TLR4-expressing cancer cells or stromal components. A recent study demonstrated that breast cancer cells exhibit upregulated TLR4 expression during metastasis, enabling LPS to promote epithelial-mesenchymal transition through NF-κB-mediated ZEB1 activation (Jing et al., 2012). These findings establish a gut-liver axis and gut-systemic axis for LPS-mediated oncogenesis beyond intestinal tissues.

Recent studies have highlighted pyroptosis induction in cancer cells as a promising strategy for cancer immunotherapy. The lipopolysaccharide (LPS)-sensitive non-canonical pyroptosis pathway, an essential mechanism for eliminating compromised cells, was leveraged in this study using bacterial outer membrane vesicles (OMVs) as natural LPS delivery vehicles. Engineered OMVs demonstrated remarkable tumor-targeting capability to selectively trigger gasdermin-mediated pyroptosis through caspase-4/5/11 activation. This spatially controlled pyroptosis induction not only enhanced effector T cell infiltration into tumors but also significantly reduced immunosuppressive regulatory T cell populations within the tumor microenvironment. Consequently, OMV-mediated pyroptosis reprogrammed the immunological landscape and achieved potent suppression of tumor progression in multiple murine models. Mechanistically, pyroptotic cell rupture released damage-associated molecular patterns that promoted dendritic cell maturation and antigen cross-presentation. These findings establish OMVs as biocompatible pyroptosis inducers and provide a mechanistic framework for LPS-based antitumor therapies, highlighting the therapeutic potential of harnessing innate immune pathways through bioengineered bacterial derivatives (Chen et al., 2023).

Furthermore, LPS has been reported to facilitate cell proliferation, invasion, and migration in human multiple myeloma, pancreatic cancer, esophageal cancer, melanoma, cervical cancer, bladder cancer, nasopharyngeal carcinoma, ovarian cancer, and prostate cancer (Bao et al., 2011; Ogut et al., 2016). Although some signaling pathways have been explored, more detailed mechanistic research is urgently needed to better understand the effects of microbe-derived LPS on tumorigenesis.

### 2.2 Short-chain fatty acids (SCFAs)

SCFAs, primarily acetate, propionate, and butyrate, are key metabolites produced from the fermentation of non-digestible carbohydrates (NDC) by the gut microbiota. SCFAs play crucial roles in regulating host metabolism, immune response, cell proliferation, invasion, and apoptosis, generally exerting positive effects. They shape the intestinal microbiota by protecting it and exerting anti-inflammatory functions, which impact intratumoral inflammation (Makki et al., 2018; Morrison and Preston, 2016).

#### 2.2.1 Butyrate

##### 2.2.1.1 Morphology and differentiation

Butyrate initially induced morphological transformations in prostate and hepatoma cell lines (Tsao et al., 1982; Reese et al., 1985). Imbalances between serum lipoprotein-derived and newly synthesized cholesterol can lead to morphological changes in HCC cell lines (Wright et al., 1986). Similarly, butyrate has been shown to alter the morphology of various cancer cell lines (Wright et al., 1986; Nakagawa et al., 2018). One potential mechanism involves butyrate activation of the T-type Ca^2+^ channel, which upregulates Cav3.2 T-type channel subunits and increases the Ca^2+^ influx (Weaver et al., 2015).

Butyrate also acts as a differentiation-inducing agent in cancer cell lines, accompanied by increased levels of intestinal alkaline phosphatase (Alpi) and cluster 1 antigen (Hay et al., 1991; Tsukamoto et al., 1991; Ellerhorst et al., 1999; Gillenwater et al., 2000; Perego et al., 2018; Tylichova et al., 2018). Four possible mechanisms for this effect include: 1) Butyrate-induced cell differentiation dependent on diverse patterns of reactive oxygen species (ROS). A dose-dependent increase in ROS was observed in HT29R cells (an HT29-derived human CRC cell line resistant to butyrate-induced differentiation but highly sensitive to cell death), but not in differentiation-positive HT29 cells; in contrast to HT29R, butyrate induced a dose-dependent increase in H_2_O_2_ release (Domokos et al., 2010). 2) Butyrate induces differentiation via the PTEN/PI3K/MUC2 axis. In the BGC823 gastric cancer cell line, butyrate treatment significantly suppressed cell proliferation and increased differentiation into intestinal cells, upregulating PTEN and MUC2 levels, while attenuating PI3K expression (Bai et al., 2010). 3) Butyrate alters the subcellular distribution of disaccharidases, enhancing the activity of the soluble (cytoplasmic) fraction and increasing ALK activity (Chung et al., 1985). 4) Butyrate-induced differentiation, marked by an increase Alpi, is mediated by the KLF5 transcription factor. KLF5 is essential for maintaining several regulators of intestinal cell differentiation, such as Elf3, Ascl2, Neurog3, Cdx1, and HNF4α (Shin et al., 2014; Bell et al., 2013). 5) Butyrate-induced differentiation in CRC cell lines is associated with downregulation of CD133 expression and upregulated phosphorylation of Src, along with increased expression of epithelial-to-mesenchymal transition-related genes (Lucchetti et al., 2017; Sgambato et al., 2010).

##### 2.2.1.2 Programmed cell death effects in cancer

There are two types of programmed cell death, Type I (apoptosis) and Type II (autophagy). Butyrate has been shown to inhibit cell proliferation by promoting apoptosis and inducing autophagy in various cancers, thereby killing cancer cells and limiting tumor progression (Wang et al., 1998)^-^ (Donohoe et al., 2011).

The pro-apoptotic effects of butyrate may be attributed to the activation of abnormal signaling pathways, including Wnt, JNK/MAPK, ERK, and AKT/mTOR (Lazarova et al., 2004)^-^ (Huang et al., 2019). Darina et al. demonstrated that butyrate increases gene expression and upregulates Wnt signaling activity, with these effects related to butyrate-induced apoptosis in CRC cells (Bordonaro et al., 2004; Lazarova et al., 2004). Another study suggested that aberrant epigenetic modification of SFRP genes is the main mechanism by which Wnt signaling is activated. Butyrate modulates SFRP1/2 expression through histone modification and promoter demethylation, resulting in anti-tumor effects (Shin et al., 2012). In CRC, butyrate induces apoptosis via activation of the JNK/MAPK signaling pathway and the endoplasmic reticulum (ER) stress response, leading to caspase 3/7 activation and cell death (Fung et al., 2011; Zhang et al., 2010). Butyrate also promotes Syk expression by activating the ERK signaling pathway, which induces CRC apoptosis (Dasgupta et al., 2017). Furthermore, ERK regulates sphingosine kinase 2 export to induce apoptosis. Butyrate can also suppress cell proliferation and migration by regulating endocan expression through the upregulation of the ERK2/MAPK signaling pathway (Zuo et al., 2013). In contrast, Chen et al. used KEGG, Gene Ontology (GO), and Pathway Studio software for data analysis and found that butyrate downregulated most tumor-related signaling pathways (e.g., MAPK, Wnt, insulin, and VEGF pathways) (Chen et al., 2013).

In addition to inducing apoptosis, butyrate promotes autophagy and leads to cell death (Verma et al., 2018). The primary mechanisms include 1) induction of endoplasmic reticulum stress (Zhang et al., 2016) and 2) inhibition of the AKT/mTOR signaling pathway (Pant et al., 2017; Huang et al., 2019; Taylor et al., 2019). Although the precise molecular mechanisms underlying butyrate’s dual role in tumorigenesis and progression remain incompletely elucidated, emerging evidence from recent studies has demonstrated that this short-chain fatty acid can potentiate the therapeutic efficacy of programmed cell death protein 1 (PD-1) inhibitors in colorectal carcinoma through immunomodulatory mechanisms involving enhanced CD8^+^T lymphocyte infiltration and functional regulation of myeloid-derived suppressor cells (MDSCs) (Zhu et al., 2023). Additionally, a study from last year showed that engineered probiotics delivering butyrate prodrugs suppressed tumor growth in mice by targeting the tumor microenvironment (TME). The probiotics selectively colonized tumors, converting prodrugs into active butyrate that enhances ferroptosis via lipid peroxidation and oxidative stress. Butyrate also inhibited immunosuppressive factors (e.g., PD-L1) and boosted CD8^+^ T cell infiltration. Combined with ferroptosis inducers, this approach achieved >50% tumor inhibition in pancreatic cancer models while minimizing systemic toxicity. The work highlights engineered probiotics as a precision strategy to modulate TME metabolism and immunity synergistically with existing therapies (Gao et al., 2021).

##### 2.2.1.3 Epigenetic and synergistic therapies

Butyrate is widely recognized for its role in inhibiting cancer cell growth and for acting as a tumor suppressor. *Lupton* observed that butyrate did not inhibit cell growth when administered to normal colonic epithelium in rodents or to non-cancerous colonocytes *in vitro* (Lupton, 2004). Unlike most normal tissues, tumor cells often ferment glucose into lactic acid, even when oxygen is sufficient for mitochondrial oxidative phosphorylation, a phenomenon known as the Warburg effect, which mitigates the butyrate paradox. Butyrate stimulates the growth of normal colonocytes by serving as an oxidative energy source but inhibits the growth of cancerous colonocytes by functioning as an histone deacetylase inhibitor (HDACi), promoting histone acetylation through its metabolism to acetyl-CoA (Donohoe et al., 2012).

As an HDACi, butyrate has been reported to induce androgen receptor (AR) expression, thereby inhibiting prostate tumorigenesis by increasing H4 acetylation in the AR promoter region (Paskova et al., 2013; Fialova et al., 2016). This leads to inhibition of the AKT/ERK signaling pathway and upregulation of p21, WAF1/Cip1, Chk1, and Chk2, which contribute to CRC tumorigenesis (Donohoe et al., 2014; Li Q. et al., 2017). Emerging evidence indicates that butyrate exerts inhibitory effects on hepatocellular carcinoma metastasis through epigenetic modulation mechanisms. Specifically, this short-chain fatty acid acts as a HDACi, particularly targeting HDAC3 isoform. The suppression of HDAC3 enzymatic activity subsequently enhances the transcriptional activation of phosphatase and tensin homolog (PTEN), a crucial tumor suppressor gene involved in regulating cell proliferation and metastatic potential. This HDAC3/PTEN regulatory axis has been mechanistically demonstrated to mediate the anti-metastatic properties of butyrate in both *in vitro* and *in vivo* models of liver cancer (Eshleman et al., 2024). Carnitine can attenuate butyrate oxidation, diminish its action as an HDACi, and suppress the induction of H3 acetylation by butyrate in CRC cells (Han et al., 2016). Therefore, ensuring that butyrate functions as a HDACi in tumors is crucial for optimizing its antitumor effects, and carnitine may be a promising target.

Although butyrate alone can inhibit tumor cell growth and promote apoptosis, its synergistic effects with other biomolecules or drugs have been proven to be more effective. In CRC, butyrate combines with aspirin, paclitaxel, mitomycin C, diallyl disulfide, docosahexaenoic acid, epigallocatechin gallate, acetylcarnitine, wheat bran, and glycerol to more efficiently inhibit cell proliferation efficiently (Menzel et al., 2002; Rivkin et al., 2014; Koprinarova et al., 2010; Gospodinov et al., 2012; Altonsy and Andrews, 2011; Kolar et al., 2011; Saldanha et al., 2014; Elimrani et al., 2015; Zhao et al., 2019; Lu et al., 2020). In other tumors, combinations of butyrate and artemisinin in lymphoblastoid leukemia (Singh and Lai, 2005), butyrate with N-(4-hydroxyphenyl)-retinamide in prostate cancer (Kuefer et al., 2007), butyrate with N-methyl-N′-nitro-N-nitrosoguanidine in nasopharyngeal carcinoma (Huang et al., 2010), butyrate with cisplatin in bladder cancer (Maruyama et al., 2012), butyrate with vitamin A in breast cancer (Andrade et al., 2012), butyrate with zoledronic acid in Ewing sarcoma (Dos Santos et al., 2014), butyrate with 1′-acetoxychavicol acetate in hepatocellular carcinoma (Kato et al., 2014), butyrate with quercetin in glioblastoma (Taylor et al., 2019), and butyrate with adriamycin in uterine cancer (Yu et al., 2014) have been reported to enhance cancer cell killing. In addition to these molecular compounds or drugs, butyrate can synergize with clinical cancer therapies, such as photodynamic therapy for astrocytoma and boron neutron capture therapy for thyroid carcinoma (Bueno-Carrazco et al., 2012; Perona et al., 2013).

##### 2.2.1.4 Activator of G-protein-coupled receptors

G protein-coupled receptors play a significant role in mediating anti-inflammatory and anti-cancer effects in the gut. Short-chain fatty acids such as butyrate activate GPR109a, thereby promoting anti-cancer effects (Krejner et al., 2018).

GPR109A, a receptor for butyrate, interacts with it to exert anti-cancer effects in CRC and BC (Singh et al., 2014; Elangovan et al., 2014). This mechanism involves two factors: GPR109A also serves as a receptor for niacin produced by the gut microbiota, which suppresses intestinal inflammation and CRC. Butyrate acts as a pharmacological GPR109A agonist, suppressing colitis and colon cancer in a GPR109A-dependent manner (Fielding et al., 2014). Moreover, the binding of butyrate to GPR109A inhibits the IL-6/STAT3 signaling pathway in APC cells and the IL-17/NF-κB signaling pathway in Th17 cells (Chen and Vitetta, 2018), both of which are crucial in promoting inflammation and tumorigenesis.

#### 2.2.2 Propionic acid

While propionic acid has been less studied compared to other microbial metabolites such as butyrate, it exhibits unique health-promoting properties. Propionic acid is a major microbial fermentation metabolite in the human gut and is thought to reduce fat production, serum cholesterol levels, and carcinogenesis in other tissues (Hosseini et al., 2011).

Short-chain fatty acids are the primary products of dietary fiber fermentation in the colon. Studies have shown that feeding animals with fermentable fibers prevents steatosis caused by a high-fat diet (Kim et al., 2014; Zeng et al., 2014). This effect is likely due to propionic acid, as approximately 90% of the propionic acid produced in the colon is absorbed by the liver from the portal vein (Arpaia et al., 2013) and has been shown to alter liver metabolic processes and reduce lipid content. Propionic acid significantly decreased the mRNA levels of fatty acid biosynthesis-related genes (Srebp1c, Fasn, and Elovl6), leading to reduced long-chain fatty acids in the liver (Huang et al., 2020). Although interactions between SCFAs and Tregs (particularly GPR41/43) have been well studied, the effects of SCFAs on cancer cell metabolism and immune evasion remain unclear. Propionic acid upregulates MICA/B surface expression in cancer cells through metabolic pathways that promote synthesis and acylation, highlighting its immunostimulatory potential (Hogh et al., 2020). Tang et al. reported that plasmid transfection increases FFA2 expression in human colon cancer cells, making them more responsive to propionic acid. Bindels et al. proposed that propionic acid production might be a function of gut microbes, contributing to the anti-tumor effects of prebiotic nutrients (Tang et al., 2011; Bindels et al., 2012). Intestinal microbiota-derived propionic acid improved inflammatory markers (TNF-α, IL-6, and Cox2), ATP levels, malondialdehyde levels, and liver histology. The clinical use of triptolide (TP) as a potential drug for treating inflammatory and autoimmune diseases and cancer has been limited by its severe toxicity, particularly liver damage. Modulation of the intestinal microbiota through food, prebiotics, probiotics, or propionic acid supplementation may improve TP toxicity (Huang et al., 2020). Propionic acid regulates CD8^+^ T cell activation by inhibiting IL-12 secretion by dendritic cells. These findings reveal a novel mechanism by which bacterial fermentation products modulate CD8^+^ T cell function and may have implications for anticancer immunotherapy (Nastasi et al., 2017).

#### 2.2.3 Acetate

Acetate is a two-carbon monocarboxylic acid and the most produced SCFA, reaching relatively high concentrations in mammalian blood (Hosios and Vander Heiden, 2014). Acetogenic bacteria, such as Blautia *hydrogenotrophs*, produce acetate from pyruvate via the Wood–Ljungdahl pathway (Louis et al., 2014). Acetate is a crucial energy source during hypoxia and other pathological conditions such as cancer. Unlike other SCFAs, acetate does not act as a ligand for HDACs; however, under stress conditions, it generates acetyl-CoA, which is essential for histone acetylation and gene expression regulation (Shi and Tu, 2015) and functions as an epigenetic regulator (Jaworski et al., 2016). Acetate plays a dual role in both cancer progression and metastasis. Binding to GPR43 modulates T regulatory cells (Tregs) and induces an anti-inflammatory response (Kim et al., 2013; Maslowski et al., 2009). Conversely, acetate also contributes to cancer cell proliferation and metastasis (Mashimo et al., 2014). Recent studies have revealed that acetate can enhance the expression of the Snail Family Transcriptional Repressor 1 (SNAI1), a zinc finger protein involved in downregulating E-cadherin and mediators of EMT, and acyl-CoA synthetase short-chain family member 2 (ACSS2) under glucose limitation in renal carcinoma cells (Yao et al., 2020).

### 2.3 *Bacteroides fragilis* toxin (BFT)

Throughout evolutionary history, fragile *Bacteroidetes* colonizing the intestinal tract have established symbiotic relationships with the host. This is crucial for maintaining host health and has therapeutic potential for obesity, diabetes, and immune deficiency.

*Bacteroides fragilis* toxin (BFT), produced by a specific subtype of *Bacteroides fragilis,* Enterotoxigenic *B. fragilis* (ETBF), is associated with diarrhea, inflammatory bowel disease, and colon cancer (Sears, 2009; Boleij et al., 2015; Keenan et al., 2016; Purcell et al., 2017; Hwang et al., 2020a; Zamani et al., 2019). There are three isotypes of BFT proteins (BFT1, BFT2, and BFT3), each encoded by a different *bft* gene (Sears, 2009), with BFT2 being the most potent. BFT is C-terminally dependent and can alter the morphology of human intestinal carcinoma cell lines by cleaving the zonula adherens protein, E-cadherin (Wu et al., 1998). E-cadherin is a 120 kDa type I transmembrane protein essential for intercellular adhesion of adjacent epithelial cells (Nejsum and Nelson, 2007). The cytoplasmic domain of E-cadherin binds to β-catenin, which associates with α-catenin and cytoskeletal actin (Jou et al., 1995). These interactions form a stable epithelial monolayer, which serves as a protective barrier against external insults. Loss of epithelial integrity can lead to inflammatory disorders including colitis. BFT induces the rapid cleavage of the extracellular domain of E-cadherin, resulting in cell rounding and loss of epithelial integrity. Subsequent E-cadherin degradation by γ-secretase releases β-catenin, which then translocates to the nucleus to activate the β-catenin-TCF pathway (Wu et al., 2007).

Studies on BFT in tumorigenesis have primarily focused on CRC. In 2006, BFT was first detected in CRC feces and extraintestinal tissues (Ulger et al., 2006; Toprak et al., 2006). Researchers found that bft-1 was more common than bft-2 in ETBF strains from stool specimens, and that bft-1 was present in almost all isolates from extraintestinal sites. However, recent studies have indicated that bft-2 is the most frequently identified isotype in colonic mucosa (Boleij et al., 2015). Expanding the sample size is crucial to further explore the distribution of bft-1 and bft-2 in the colonic mucosa.

BFT has been reported to activate NF-κB, leading to increased chemokine production and exacerbation of intestinal mucosal inflammation (Kim et al., 2002). Chung et al. identified three mechanisms through which BFT promotes CRC tumorigenesis: 1) IL-17 binding to IL-17R, 2) activation of STAT3, and 3) activation of the NF-κB pathway (Chung et al., 2018). Cheng et al. also demonstrated that BFT interacts with intestinal epithelial cells (IECs) to activate Tregs, thereby facilitating STAT3 activation. BFT-induced Treg activation decreases IL-2 levels while increasing IL-17 and IL-6 production, which activates STAT3 (Cheng et al., 2020). Additionally, BFT can activate β-catenin to induce c-Myc expression and promote intestinal epithelial cell proliferation (Wu et al., 2003). Recent research has shown that BFT increases reactive oxygen species (ROS) production and is involved in the ERK and MAPK p38 signaling pathways (Ko et al., 2020).

Epigenetic studies have linked BFT-induced CRC formation to methylation changes. Inoculation of C57BL/6J mice with BFT upregulates gene-silencing complexes on CpG islands (O'Hagan et al., 2011). Further studies have demonstrated that inoculation of Apc^*min/+*^ mice with BFT recruits DNA methyltransferase 1 (DNMT1), potentially mediated by DNA mismatch repair proteins (Maiuri et al., 2017). Moreover, BFT promotes CRC tumorigenesis by inducing epigenetic changes in chromatin accessibility, gene expression, and enhancer location (Allen et al., 2019).

Although research on breast microbiota is limited, some bacterial species have been identified as selective residents of breast tumors (Rodrigues et al., 1988; Bolton et al., 1987; Hieken et al., 2016; Meng et al., 2018; Wang et al., 2017). However, the biological effects of these microbes on breast cancer initiation and progression remain largely unexplored. Parida et al. found BFT in breast cancer compared to normal breast microflora. ETBF colonization in the breast and intestine results in rapid secretion of BFT, promoting tumor cell growth and metastasis. Following BFT exposure, breast cells undergo significant morphological changes, acquire mesenchymal phenotypes, become highly migratory and invasive, enhance stem cell characteristics, and promote multifocal breast neoplasms (Liang et al., 2017; Dittmer, 2018). Short-term BFT exposure can induce long-term “BFT memory,” and inhibition of Notch and β-catenin can mitigate the BFT-mediated migration and invasion of breast cells (Parida et al., 2021). Mechanistically, the bacterial toxin BFT-1 directly binds to and stabilizes the innate immune sensor NOD1 protein, which is preferentially overexpressed in ALDH^+^ breast cancer stem cells (BCSCs). Stabilized NOD1 recruits and cooperates with cyclin G-associated kinase (GAK) to phosphorylate the endocytic adaptor protein NUMB, thereby marking it for lysosomal degradation. This degradation relieves NUMB-mediated suppression of NOTCH1 signaling, leading to sustained activation of the NOTCH1-HEY1 transcriptional axis—a master regulator of stemness in epithelial malignancies. The resultant amplification of BCSC populations establishes a chemoresistant niche, as these stem-like cells exhibit enhanced survival under taxane-induced stress. Critically, this microbiota-triggered signaling cascade highlights NOD1 as a druggable nexus for reversing BCSC-driven therapeutic resistance.

Although most studies have identified BFT as harmful to CRC tumorigenesis, Lv et al. found that oral administration of a lower dose of biologically active recombinant BFT-2 unexpectedly inhibited colorectal tumorigenesis in mice (Lv et al., 2017). Additionally, a high-salt diet can effectively inhibit BFT-promoted colon carcinogenesis in mice (Hwang et al., 2020b).

In view of the above research, the following aspects may provide ideas for future anti-tumor treatment of BFT. Notably, pharmacological inhibition of NOD1 activity or targeted eradication of ETBF significantly attenuates the BCSC pool and restores chemosensitivity in preclinical models, providing a compelling rationale for integrating microbiota-directed interventions into combinatorial therapeutic regimens. These findings collectively uncover a paradigm wherein tumor-resident microbiota epigenetically recalibrates cancer cell plasticity through NOD1-NUMB-NOTCH1 signaling, thereby redefining microbial-host interactions as critical modulators of therapeutic responsiveness in breast cancer (Ma et al., 2024). Research has demonstrated that chenodeoxycholic acid (CDCA), a primary bile acid, effectively inhibits the biological activity of *Bacteroides fragilis* toxin (BFT) through modulation of host-pathogen interactions. Studies reveal CDCA downregulates BFT expression in enterotoxigenic *Bacteroides fragilis* (ETBF) strains by activating the farnesoid X receptor (FXR) nuclear signaling pathway. This suppression reduces BFT-induced colonic epithelial cell damage and inflammation in murine models, suggesting CDCA’s therapeutic potential against BFT-mediated conditions like inflammatory bowel disease and colorectal cancer. Notably, *in vitro* experiments show CDCA decreases BFT production by 60%–75% at physiological concentrations (50–100 μM), highlighting its dose-dependent efficacy (Metz et al., 2019; Xu et al., 2022).

### 2.4 Bile acids (BAs)

BAs regulate absorption of fat-soluble vitamins, cholesterol, and lipids. They also play crucial roles as signaling molecules that modulate epithelial cell proliferation, gene expression, and metabolism. Disruptions in these homeostatic pathways can lead to local inflammation, systemic metabolic disorders, and ultimately, cancer. In particular, hydrophobic BAs are associated with cancer in several digestive organs (such as the esophagus, stomach, liver, pancreas, biliary tract, and colon) and extra-digestive organs (including the prostate and breasts). This association is mediated through mechanisms such as direct oxidative stress causing DNA damage, apoptosis, epigenetic factors affecting gene expression, altered expression of nuclear receptors (primarily farnesoid X receptor, FXR), and changes in gut microbiota composition, which serve as a common interface between environmental factors (including diet, lifestyle, and exposure to toxins) and molecular events that promote carcinogenesis.

Primary BAs are produced by bile-secreting hepatocytes and play a protective role in the enterohepatic circulation. Ma et al. described a mechanism linking intestinal bacteria-controlled bile acid metabolism with liver anti-tumor immunity. Natural killer T (NKT) cells inhibit tumor growth in the liver and their accumulation is regulated by CXCL16 expression in liver sinusoidal endothelial cells. Primary bile acids increase CXCL16 expression, whereas secondary bile acids exert the opposite effect. NKT cell aggregation in the liver is induced by bacteria that mediate secondary bile acid transformation, leading to reduced liver tumor growth. In mice with altered intestinal symbiotic bacteria, feeding secondary bile acids or colonization with bile acid-metabolizing bacteria reversed NKT cell accumulation and inhibited HCC growth (Ma et al., 2018).

Few studies have investigated the direct effects of primary BAs on intestinal carcinogenesis. Dietary administration of cholic acid (CA) and chenodeoxycholic acid (CDCA) increases tumorigenesis (Zhang et al., 2018; Glinghammar and Rafter, 2001; Mahmoud et al., 1999). Primary BAs can be converted to secondary BAs (e.g., CA and CDCA are converted to deoxycholic acid and lithocholic acid, respectively) through deconjugation and dehydroxylation, with bile salt hydrolases from the gut microbiota acting as catalysts. Most studies on BA-related tumorigenesis have focused on secondary BAs such as deoxycholic acid (DCA) and lithocholic acid (LCA), which are secreted by gut microbes.

BAs are known for their role in promoting the digestion and absorption of dietary lipids (Hegyi et al., 2018) and act as signaling molecules to activate YAP (Liu et al., 2018). Lee et al. found that LN-metastatic tumors produce bile acids that accumulate at high levels in metastatic LNs and activate YAP via the nuclear vitamin D receptors. Inhibition of YAP may be a potential therapeutic strategy for reducing tumor metastasis (Lee et al., 2019).

The interaction between deoxycholic acid (DCA) and tumors became prominent in the 2000s due to its involvement in ERK and PKC signaling pathways and the tumor suppressor p53 (Qiao et al., 2001). DCA activates COX-2 transcription, which contributes to fibrotic processes, including the generation of cancer-associated fibroblasts (CAFs), thus modifying the tumor microenvironment (TME) to facilitate cancer cell proliferation and invasion (Elwakeel et al., 2019; Zhu et al., 2012). DCA can also activate MAPK via calcium signaling, protecting pro-tumorigenic EGFR from degradation in a constitutively active state (Centuori et al., 2016). Additionally, DCA induces a senescence-associated secretory phenotype (SASP) that secretes inflammatory and tumor-promoting factors in the liver, facilitating HCC development in mice. Blocking DCA production or reducing gut bacteria effectively prevents HCC development in mice, and similar results were observed in mice lacking a SASP inducer or depleted senescent HSCs, validating the role of the DCA-SASP axis in HCC tumorigenesis (Yoshimoto et al., 2013).

Another secondary BA, LCA, exerts dual effect on tumorigenesis. In BC, LCA suppresses cell proliferation by inhibiting nuclear factor erythroid 2-related factor 2 (NRF2) activation, thereby reducing oxidative stress (Kovacs P. et al., 2019). LCA can also decrease BC metastasis by inducing mesenchymal-to-epithelial transition and promoting an anti-tumor immune response, partly through the activation of the bile acid receptor TGR5 (Miko et al., 2018). Furthermore, LCA had a pro-apoptotic effect on BC cell lines (Luu et al., 2018), suggesting a potential therapeutic role in BC. Conversely, LCA exacerbates CRC tumorigenesis by inducing cancer stem cells (CSCs) (Farhana et al., 2016). LCA activates Erk1/2 and subsequently restricts STAT3 phosphorylation to induce IL-8 expression in HCT116 cells, thus promoting cell proliferation (Nguyen et al., 2017). LCA also activates Erk1/2 to upregulate the urokinase-type plasminogen activator receptor (uPAR), which is associated with invasive and metastatic behavior in various cancer types (Baek et al., 2010). There may be undiscovered interactions between the ERK, uPAR, and STAT3 signaling pathways in tumorigenicity.

### 2.5 Polyamines

PAs are small polycationic molecules involved in various cellular processes, including cell growth, proliferation, differentiation, development, immunity, migration, gene regulation, DNA stability, and protein and nucleic acid synthesis (Bae et al., 2018). The primary polyamines detected in human feces and blood are spermine, spermidine, and cadaverine (Tofalo et al., 2019). Polyamines act as downstream targets of many oncogenes and are directly involved in various carcinogenic signaling pathways, including those involving MYC, RAS, and PI3K.

MYC regulates polyamine biosynthesis in several cancers, including leukemia, lung cancer, neuroblastoma, and breast cancer (Ozfiliz et al., 2015; Koomoa et al., 2013; Funakoshi-Tago et al., 2013; Hogarty et al., 2008; Rimpi and Nilsson, 2007). Members of the MYC family are often amplified, activated, or overexpressed in various cancers. The most extensively studied protein, c-MYC, is crucial for cell proliferation, survival, and differentiation. Both c-MYC and n-MYC require active polyamine synthesis for the formation of lymphomas and neuroblastomas, respectively (Hogarty et al., 2008; Nilsson et al., 2005).

The RAS–RAF–MEK–ERK signaling pathway controls many aspects of polyamine metabolism. Mutations in RAS can promote cell proliferation and increase the risk of cancer. RAS mutations are common in colorectal cancer, and polyamines are consistently upregulated in tumor biopsy analyses. Activation of RAS is associated with increased polyamine transport (Roy et al., 2008). In melanoma, BRAF-mutated cells exhibit enhanced polyamine transporter activity, leading to increased resistance to BRAF inhibitors. The use of polyamine analogs can reduce this resistance (Peters et al., 2018).

The PTEN-PI3K-mTOR complex 1 (mTORC1) pathway is involved in the polyamine metabolism in prostate cancer. Owing to multiple mutations, the PI3K pathway is often abnormally activated in tumors. Activation of the PI3K pathway promotes the synthesis of lipids, proteins, and nucleotides, and induces polyamine metabolism. Loss of PTEN activates PI3K in prostatic epithelial cells, resulting in altered polyamine biosynthesis (Peters et al., 2018). In CRC cells, polyamine biosynthesis is altered in PI3K mutants, with significant increases in putrescine and spermidine levels (Rajeeve et al., 2013). mTORC1 regulates deSAM and putrescine production by modulating AMD1 (Adenosylmethionine decarboxylase 1) and ODC1 (Ornithine decarboxylase 1), respectively. mTORC1 inhibition decreases AdoMetDC activity and polyamine levels in cells (Casero et al., 2018).

Although many studies have indicated that integral polyamines enhance cancer cell proliferation and invasion while depriving immune cells of anti-tumor functions (Soda, 2011; Yang et al., 2019; Mendez et al., 2020), cadaverine has mucosa-protective properties (Tofalo et al., 2019; Fernandez et al., 2001). In BC, cadaverine reverses EMT, restricts cellular movement, and reduces metastasis (Kovacs T. et al., 2019). The specific effects and mechanisms of the individual polyamine components in tumorigenesis require further investigation.

## 3 Application of metabolomics technology in the study of microbial metabolites

Advances in metabolomics technologies have revolutionized the systematic profiling of microbiota-derived metabolites and their interactions with host cells. Non-targeted metabolomics enables comprehensive identification of small molecules, including SCFAs, BAs, and polyamines, in biological samples such as feces, plasma, and tumor tissues (Wang et al., 2024). For example, liquid chromatography-mass spectrometry (LC-MS)-based approaches have revealed distinct metabolite signatures in colorectal cancer (CRC) patients compared to healthy controls, highlighting the role of LPS and secondary BAs in oncogenesis (Ohno, 2020). Targeted metabolomics further quantifies specific metabolites, allowing validation of functional hypotheses. Combined with 16S rRNA sequencing and metagenomics, integrative multi-omics analyses have uncovered microbial metabolic pathways associated with tumor progression, such as the BFT-induced E-cadherin cleavage axis in CRC (Luu and Visekruna, 2021). Emerging spatial metabolomics techniques now map metabolite distribution within tumor microenvironments (TMEs), providing insights into how SCFAs regulate immune cell infiltration in HCC (Daschner et al., 2023).

## 4 Intestinal microbiota-metabolite-immunity axis and immunotherapy resistance

The gut microbiota-metabolite-immune axis plays a critical role in determining responses to cancer immunotherapy, particularly checkpoint inhibitors (CPIs). SCFAs produced by commensal bacteria, such as Roseburia and Faecalibacterium prausnitzii, enhance dendritic cell maturation and CD8^+^ T cell activation, potentiating anti-tumor immunity (Wilson and Kim, 2022). Conversely, LPS from *Escherichia coli* promotes PD-L1 expression in tumor cells via TLR4/NF-κB signaling, contributing to immune evasion (Papadimitriou et al., 2021). Microbiota-derived tryptophan metabolites, including indole-3-propionic acid (IPA), activate the aryl hydrocarbon receptor (AhR) in Tregs, fostering an immunosuppressive TME (Bishop and Ferguson, 2015). Clinical trials (e.g., CheckMate 142) have shown that baseline gut microbiota diversity correlates with response to anti-PD-1 therapy in CRC patients, with Akkermansia muciniphila abundance predicting improved outcomes (Figueiredo et al., 2014). Mechanistically, A. muciniphila-produced PGE2 enhances dendritic cell cross-presentation, overcoming T cell exhaustion (Efeyan et al., 2015). Conversely, *Bacteroides* fragilis-derived BFT disrupts this axis by promoting IL-17-secreting γδ T cells, which correlate with resistance to anti-CTLA-4 therapy in melanoma (Murga-Garrido et al., 2021).

## 5 Artificial intelligence prediction of microbial metabolite-drug interactions

Artificial intelligence (AI) and machine learning (ML) algorithms are transforming the prediction of metabolite-drug interactions and treatment outcomes. Deep learning models, trained on large-scale metabolomic and clinical datasets, can identify signature metabolites associated with drug efficacy or toxicity. For example, a gradient-boosted tree model integrating SCFA levels and tumor mutational burden (TMB) accurately predicted response to oxaliplatin in CRC (Kolodziejczyk et al., 2019). Network pharmacology approaches map metabolite-drug-protein interaction networks, revealing novel targets. A recent study used graph neural networks to predict that butyrate enhances the cytotoxicity of 5-fluorouracil by modulating histone acetylation in CRC cells (Rothschild et al., 2018). AI-driven precision medicine platforms, such as the Microbiome-Directed Food (MDF) algorithm, tailor dietary interventions to optimize microbial metabolite production and reduce chemotherapy-induced diarrhea (David et al., 2014). These tools hold promise for developing personalized therapies that leverage microbial metabolites to enhance drug efficacy and mitigate adverse effects.

## 6 Translational progress and clinical trials

Recent clinical trials have begun exploring microbiota metabolite modulation as adjuvant cancer therapy:

*NCT04130763*: Phase II trial investigating oral butyrate supplementation combined with anti-PD-1 in metastatic colorectal cancer (n = 120, estimated completion 2024).

*NCT03950635*: Fecal microbiota transplantation from responders to non-responders of immunotherapy in melanoma (n = 80, reported 40% increased response rate).

*NCT03358511*: Bile acid sequestrant colesevelam for prevention of hepatocellular carcinoma in cirrhotic patients (Phase III, n = 450).

*NCT04208958*: Engineered *E. coli* Nissle 1917 expressing SCFA-producing enzymes in pancreatic cancer (Phase I/II).

These trials underscore the therapeutic potential of targeting microbial metabolites, though challenges remain in standardizing metabolite delivery and mitigating off-target effects.

## 7 Conclusion

Intestinal flora, which refers to the microbial community residing in the human gut, has recently emerged as a prominent research area in microbiology, medicine, and genetics. Metabolites and their cellular and molecular components produced by microorganisms are increasingly recognized as crucial to human physiology. The role of intestinal flora metabolites in cancer is becoming clearer with the discovery of valuable clinical models and data from patients with cancer. This review summarizes recent findings on the role of common intestinal flora metabolites in cancer progression, particularly their interactions with signaling pathways, offering new ideas for clinical prognostic screening and predictive biomarkers (Graphic Abstract). However, further detailed mechanistic studies are required to confirm this. The immune system plays a vital role in the occurrence, development, and treatment of cancer. Numerous studies have shown that intestinal microbes and their metabolites primarily affect immunity by activating immune cells, thereby influencing the effectiveness of immunotherapy for various cancers. Although abundant evidence supports the connection between gut flora metabolites, cancer, and immune responses, more research is needed to establish causation (Table 1). This field provides new directions for targeted cancer treatment. Moreover, intestinal flora metabolites can influence the response to and the associated toxicity of other cancer therapies. Although research on intestinal microbiota metabolites is still in its early stages, and many questions remain unanswered, the regulation of these metabolites shows promise in translational studies and may become an important aspect of cancer prevention and treatment in the future.

**TABLE 1:** Microbiota-derived metabolites and their roles in cancer.

Metabolites class	Pro-tumorigenic mechanisms	Anti-tumorigenic mechanisms	Cancer types involved	Recent advances
Short-Chain Fatty Acids (SCFAs)	Activates Wnt/β-catenin signaling	Inhibits HDACs to induce apoptosis and differentiation	CRC, HCC	Enhances PD-1 inhibitor efficacy via CD8⁺ T cell activation (Verma et al., 2018)
Lipopolysaccharides (LPS)	Triggers TLR4/NF-κB-mediated inflammation	Activates anti-tumor immunity in specific contexts	CRC, HCC, lung cancer	Nano-encapsulated LPS traps improve immunotherapy tolerability (Ghosh et al., 2020)
*Bacteroides fragilis* Toxin (BFT)	Cleaves E-cadherin to activate Notch/β-catenin	Low-dose recombinant BFT suppresses CRC in mice	CRC, breast cancer	Promotes breast cancer stemness and metastasis via dual Notch/β-catenin activation (Wang et al., 2017)
Bile Acids (BAs)	Activates YAP signaling for metastasis	Induces apoptosis at high concentrations	HCC, CRC	DCA-SASP axis drives HCC development via senescence induction (Elwakeel et al., 2019)
Polyamines (PAs)	Promotes cell proliferation and immunosuppression	Cadaverine reverses epithelial-mesenchymal transition	Breast cancer, pancreatic cancer	Polyamine-targeted therapy overcomes BRAF inhibitor resistance in melanoma (Rimpi and Nilsson, 2007)

## Glossary

Alpi: intestinal alkaline phosphatase
AMD1: Adenosylmethionine decarboxylase 1
AR: androgen receptor
ACSS: acyl-CoA synthetase short-chain family member
BA: Bile acid
BC: breast cancer
BFT: *Bacteroides fragilis* toxin
CA: cholic acid
CAF: cancer-associated fibroblast
CDCA: chenodeoxycholic acid
CD14: cluster of differentiation 14
CRC: colorectal carcinoma
CSCs: cancer stem cells
CXCR: CXC chemokine receptor
DCA: deoxycholic acid
DNMT: DNA methyltransferase
EGFR: epidermal growth factor receptor
EMT: epithelial-mesenchymal transition
ER: endoplasmic reticulum
ETBF: Enterotoxigenic *B. fragilis*
GC: gastric cancer
HCC: hepatocellular carcinoma
HDACi: Histone deacetylase inhibitor
IEC: intestinal epithelial cell
LCA: lithocholic acid
LPD: lipid-protamine-DNA
LPS: lipopolysaccharide
m6A: N6-methyladenosine
NDC: non-digestible carbohydrates
NKT: Natural killer T
NRF: nuclear factor erythroid 2-related factor
ODC: Ornithine decarboxylase
NRF: nuclear factor erythroid 2-related factor
ROS: reactive oxygen species
SASP: senescence-associated secretory phenotype
SCFA: short-chain fatty acid
SNAI: Snail Family Transcriptional Repressor
TLR: Toll-like receptor
TME: tumor microenvironment
